# Assessing supervisor versus trainee viewpoints of entrustment through cognitive and affective lenses: an artificial intelligence investigation of bias in feedback

**DOI:** 10.1007/s10459-024-10311-9

**Published:** 2024-02-23

**Authors:** Brian C. Gin, Olle ten Cate, Patricia S. O’Sullivan, Christy Boscardin

**Affiliations:** 1 Department of Pediatrics, University of California San Francisco, 550 16th St Floor 4, UCSF Box 0110, San Francisco, CA 94158 USA; 2 Utrecht Center for Research and Development of Health Professions Education, University Medical Center, Utrecht, the Netherlands; 3 Department of Medicine, University of California San Francisco, San Francisco, USA; 4 Department of Surgery, University of California San Francisco, San Francisco, USA; 5 Department of Anesthesia, University of California San Francisco, San Francisco, USA

**Keywords:** Entrustment, Feedback, Clinical supervision, Gender bias, Natural language processing, Large language models, Artificial intelligence

## Abstract

The entrustment framework redirects assessment from considering only trainees’ competence to decision-making about their readiness to perform clinical tasks independently. Since trainees and supervisors both contribute to entrustment decisions, we examined the cognitive and affective factors that underly their negotiation of trust, and whether trainee demographic characteristics may bias them. Using a document analysis approach, we adapted large language models (LLMs) to examine feedback dialogs (N = 24,187, each with an associated entrustment rating) between medical student trainees and their clinical supervisors. We compared how trainees and supervisors differentially documented feedback dialogs about similar tasks by identifying qualitative themes and quantitatively assessing their correlation with entrustment ratings. Supervisors’ themes predominantly reflected skills related to patient presentations, while trainees’ themes were broader—including clinical performance and personal qualities. To examine affect, we trained an LLM to measure feedback sentiment. On average, trainees used more negative language (5.3% lower probability of positive sentiment, *p* < 0.05) compared to supervisors, while documenting higher entrustment ratings (+ 0.08 on a 1–4 scale, *p* < 0.05). We also found biases tied to demographic characteristics: trainees’ documentation reflected more positive sentiment in the case of male trainees (+ 1.3%, *p* < 0.05) and of trainees underrepresented in medicine (UIM) (+ 1.3%, *p* < 0.05). Entrustment ratings did not appear to reflect these biases, neither when documented by trainee nor supervisor. As such, bias appeared to influence the emotive language trainees used to document entrustment more than the degree of entrustment they experienced. Mitigating these biases is nonetheless important because they may affect trainees’ assimilation into their roles and formation of trusting relationships.

**Supplementary Information:**

The online version contains supplementary material available at 10.1007/s10459-024-10311-9.

## Introduction

While educators have widely adopted entrustment frameworks in assessment, the effects of these implementations on trainee learning are only beginning to be understood. Intuitively, entrustment should support trainee learning and professional growth by affording them an optimal balance between supervision and autonomy (ten Cate et al., 2016). Entrustment operationalizes this balance via decisions that rely on a supervisor’s trust in a trainee to perform clinical tasks with varying levels of independence, and encourages feedback on the competencies needed for progressive independence. The competencies, qualities, and behaviors that a trainee may demonstrate to gain their supervisor’s trust have been examined closely from the supervisor standpoint (Dijksterhuis et al., 2009; Hauer et al., 2013; Kennedy et al., 2007; ten Cate & Chen, 2020), and to a lesser extent from the trainee standpoint (Caro Monroig et al., 2021; Gin et al., 2021; Karp et al., 2019). While supervisor and trainee perspectives do mirror each other with respect to the general scope of factors related to earning trust, it is less clear whether trainees respond both cognitively and affectively to entrustment decisions as their supervisors intend (Martin et al., 2020). Regarding cognition, it is unclear if trainees regard the same factors as equally important as their supervisors do for earning clinical trust. Regarding affect, it is unclear whether trainee emotional responses elicited by their supervisors’ entrustment decisions serve to further their learning, or if they may unintentionally reinforce biases in the clinical learning environment. Providing clarity on the cognitive and affective states of supervisors and trainees surrounding entrustment decisions—and identifying potential biases that can shape them—are thus key to developing supervisor-trainee relationships that lead to assessment *for* learning (AfL) and ensuring equitable implementation of entrustment.

Feedback dialogs around entrustment-granting clinical encounters can provide a window into the cognitive and affective states of both supervisors and trainees in the negotiation of trust. With respect to cognition, such feedback should reflect factors supervisors consider when making entrustment decisions, including both trainees’ competence and personal qualities (Gin et al., 2022). Several studies examined the factors that supervisors and trainees view as important for earning trust, but separately. From the supervisor standpoint, studies have focused on factors influencing supervisors’ decisions to entrust trainees. Theoretical studies developed five factors that supervisors consider (trainee, supervisor, context, task, and relationship) (Dijksterhuis et al., 2009; Hauer et al., 2013; Holzhausen et al., 2017; Kennedy et al., 2007), which were supported by empiric studies primarily based on retrospective supervisor interviews (Hauer et al., 2015; Nelson et al., 2023; Sheu et al., 2016). More recently ten Cate and Chen (2020) developed a framework that summarizes trainee qualities for entrustment found in the literature. While trainees are aware that these factors influence their supervisors’ trust in them (Gin et al., 2021; Karp et al., 2019), supervisors with a performance focus may base their assessments on how well trainees demonstrate clinical competencies, while trainees adapting to the clinical learning environment may be more attuned to how their developing roles and relationships can act as gatekeepers to participation (Caro Monroig et al., 2021; Castanelli et al., 2021, 2022; Hatala et al., 2022; Pugh & Hatala, 2016). Such differences could be investigated by exploring the thematic content of supervisors’ and trainees’ documentation of feedback dialogs about similar types of clinical tasks, in similar contexts. Furthermore, such feedback may reflect actual supervisor decisions occurring in practice, as compared to interviews reflecting aggregate experiences retrospectively.

Emotion may be a key element that shapes trainees’ prioritization of entrustment-determining factors. As trainees often face unfamiliar (and sometimes uncomfortable) clinical learning environments, it can be overwhelming for a trainee to manage all factors that could potentially influence their supervisor’s trust in them (Martin et al., 2020). Such prioritization is thought to not only involve cognitive, but also affective processes. In the psychology literature, emotion has been conceptualized as a lens that modulates one’s prioritization of the cognitive tasks at hand (Simon, 2020). In the health professions education literature, emotion has been demonstrated to be linked to trainees’ feedback receptivity (Cordovani et al., 2023; Mills et al., 2023), and supervisors’ willingness to entrust (Gomez-Garibello & Young, 2018). Emotions reflected specifically in narrative data have also been investigated. Prior work on feedback by Ginsburg et al. (2016) utilized the lens of politeness theory to demonstrate that social pressures to maintain effective supervisor-trainee relationships led to a lack of directness in the tone used by supervisors. Feedback documented by trainees may not reflect such pressures when directed towards themselves. Trainees were found to make active decisions about whether to accept feedback, based on their judgement of the credibility of the feedback provider (van de Ridder et al., 2015). If a trainee were to document a supervisor’s feedback that they did not agree with, the language that the trainee uses may reflect ambiguity or a lack of agency, since the emotional content of language can reflect a trainee’s perceptions of competence and self-efficacy (Sagasser et al., 2017). Assessing the emotions reflected by narrative text can be performed using a technique called sentiment analysis (Tausczik & Pennebaker, 2010). Such an analysis on supervisor and trainee documentation of feedback dialogs may thus provide insight into the affective processes that influence trust.

A study of the cognitive and affective processes affecting supervisor and trainee experiences of entrustment would not be complete without also considering potential biases that may affect them. Given trust’s dependence on human judgement and instinct (i.e. “swift trust” that is based upon little data or experience) (ten Cate et al., 2016), these viewpoints are inevitably susceptible to bias. These biases may relate to trainee demographic characteristics—such as gender or underrepresented in medicine (UIM) status—and may reinforce detrimental affective states leading to assessments with negative consequences on trainee development (Hauer et al., 2023; Rojek et al., 2019; Teherani et al., 2018). Multiple junctures within entrustment are subject to bias, including: the entrustment ratings themselves, the content of the narratives, and the language (e.g. sentiment) used in the narratives. Studies examining bias in entrustment and feedback have found conflicting evidence. Recently, Padilla et al. (2022) examined entrustment ratings in a surgical residency context for gender bias. They found no such bias in assessments submitted by faculty, but a negative bias in self-assessments submitted by female residents. Dayal et al. (2017) examined milestone ratings in an emergency medicine residency, finding a bias in the rate of milestone attainment that favored male residents. With respect to content, Mamtani et al. (2022) performed a large qualitative study comparing feedback themes in narrative comments given to male and female residents in an emergency medicine setting, finding that female residents were more likely to be told they lacked confidence with procedural skills. Rojek et al. (2019) examined adjectives used in medical students’ clinical evaluations, finding biases related to both students’ gender and under-represented minority (URM) status. Similar biases in feedback content and sentiment related to trainee gender have either been suggested (but found to lack statistical significance) (Minter et al., 2005) or found to be unlikely (Andrews et al., 2021).

While qualitative studies led to retrospective insights about supervisor and trainee viewpoints of entrustment, and quantitative studies found conflicting results on bias in different settings, systematic analysis of a large dataset of entrustment-associated narratives may allow us to perform both analyses simultaneously and assess how they interact. A large dataset taken from an institution-wide experience over several years may thus allow for identification of systematic differences in trainee and supervisor viewpoints, and examination of potential biases represented in the narratives. However, performing consistent text analysis of this nature across a large dataset would be difficult to do via manual coding, and may be prone to bias of the coders themselves. Recently, the development of large language models (LLMs) has facilitated innovations in natural language processing (NLP) and artificial intelligence (AI) that elevate the ability of NLP to characterize themes and emotions (Alaparthi & Mishra, 2021; Boscardin et al., 2023; Zhang et al., 2023). LLMs underly generative AI applications such as ChatGPT, Claude, and Bard. LLMs are implemented via artificial neural networks that have been trained to represent language probabilistically, considering interrelationships of words in the context of sentences, paragraphs, bodies of text, and entire corpora. When applied to narrative excerpts, they can be used for many NLP applications, including: representing meaning numerically (i.e. via embeddings), producing specific output (i.e. measuring sentiment), and generating new next based on specific prompts (i.e. chatbots). In these applications, LLMs carry a significant advantage in semantic fidelity over traditional NLP methodologies that derive mostly from word frequency (Rojek et al., 2019; Tausczik & Pennebaker, 2010) rather than higher levels of meaning (Boscardin et al., 2023).

In this study we developed and utilized NLP tools based on LLMs to systematically compare the thematic content and sentiment of feedback dialogs from observed clinical encounters documented either by supervisors or trainees. We developed a gender-neutral sentiment analysis strategy to mitigate algorithmic bias. By examining how supervisors and trainees documented such feedback dialogs on an institution-wide scale over two years, we quantitatively compared the cognitive and affective factors shaping their interpretation of entrustment-related feedback as revealed by: (1) the thematic content used to justify entrustment ratings, (2) the sentiment of the language used, and (3) the susceptibility of entrustment ratings and sentiment to potential sources of bias, including gender and UIM status.

## Methods

### Positionality and overview of AI-assisted document analysis

We considered feedback dialogs to be co-constructed through the interaction of supervisors and trainees (Dudek et al., 2019; Telio et al., 2016)—potentially shaped by biases tied to demographic characteristics of trainees (Andrews et al., 2021; Herrenkohl et al., 2022). Interactions between supervisors and trainees included both the clinical observation, the clinical performance of the trainee, any corrective or reinforcing actions taken by the supervisor, and the feedback dialog occurring afterwards. While both parties participated in each of these steps, we hypothesized that documentation of these interactions would reveal differences in the perspectives of supervisors and trainees when written separately by either participant. Thus, we adopted a document analysis approach to evaluate these feedback narratives as a retrospective analysis of existing assessment data (Cleland et al., 2023). Our analysis focused on the content (themes related to entrustment ratings), linguistic character (sentiment), and latent content (the trainee’s implied acceptance or rejection of feedback, and evidence of influence from sources of bias) of the documents.

**Fig. 1:**
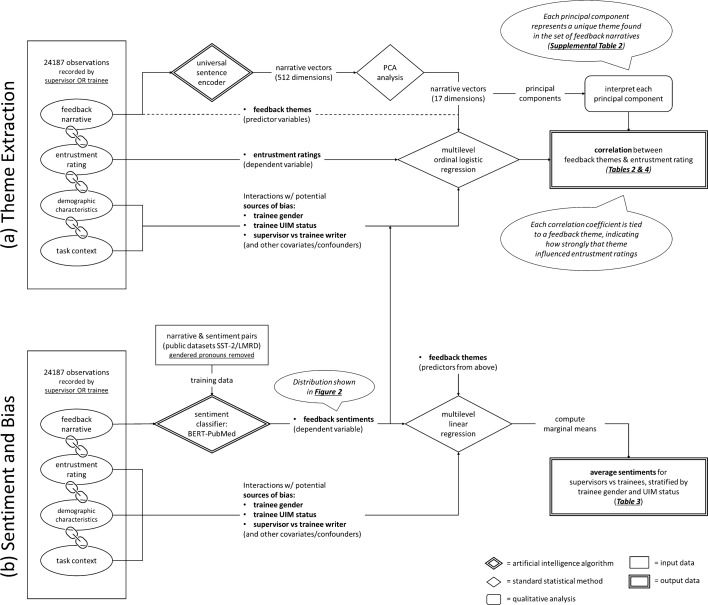
Outline of AI-assisted **a** theme extraction, and **b** sentiment and bias analysis

We developed AI strategies as extensions of analytic procedures we would have performed manually using a reflexive thematic analysis approach (Braun & Clarke, 2021), had the dataset been orders of magnitude smaller. An overall outline of the strategy is shown in Fig. 1.

- *For theme extraction* We developed a transfer learning^1^ AI approach based on a previously-trained LLM to abstract the thematic content of each individual narrative numerically, and used principal component analysis (PCA) to segregate the space of relevant content into its most prominent principal components. During this process, we employed a panel of expert coders to iteratively define and refine the themes these principal components represented, as would be done in traditional qualitative coding.
- *For sentiment analysis* We trained an LLM to classify narratives by their probability of having a negative or positive emotional valence.
- *For investigation of bias* To look for evidence of bias in the narrative dataset, we first had to assess the LLM’s own bias towards gender, and developed a strategy to mitigate this bias by removing gender-associated pronouns and nouns from both training, validation, and study datasets.
- *For statistical analysis* We performed our final statistical analysis using standard multilevel modeling to account for the nested structure of the data (multiple entries associated with each individual student), including investigation of important confounders and covariates.

We refined our data analysis and algorithms iteratively during the process of data collection, but we did not alter the instrument or scope of data collection during the study. We have made all source code we used and developed for data analysis publicly available at the link in the footnote.^2^

### Data, participants, and setting

The data consisted of feedback narratives generated from N = 24,187 discrete feedback dialogs between 552 clerkship-year medical student trainees and their direct clinical supervisors (4926) that occurred following observed clinical tasks (physical exams, history-taking, procedures, note-writing, communications, oral presentations, and other) (Table 1). The narrative from each feedback dialog was documented either by the supervisor or trainee, but not by both. The documentation also included a rating (from 1 to 4, see Supplemental Table S1) of the level of supervision provided for the clinical encounter (based on the Modified O-SCORE scale), which we refer to here as the *entrustment rating* (ten Cate et al., 2020). While both the feedback narrative and entrustment rating prompts were adjacent to each other on the assessment instrument, the narrative prompt did not explicitly require elaboration on why a particular entrustment rating was chosen. The instructions and data collection instrument were identical for both supervisors and trainees. The data were collected over two calendar years (January 2020–December 2021) from every clerkship-year medical student in all required clinical clerkships at a 4-year post-baccalaureate medical school in the United States. Students were asked to complete two such observations weekly during their required clerkships (which included: pediatrics, internal medicine, obstetrics/gynecology, neurology/psychiatry, surgery, family/community medicine, and anesthesia). Identities of both students and supervisors were masked and replaced with random tokens.

**Table 1:** Characteristics of the assessment dataset, collected from Jan 2020 to Dec 2021 of all clerkship-year medical students across a single US-based medical school

Total number of students	552
Male gender (%)	256 (46%)
Female gender (%)	293 (53%)
Neither male/female gender (%)	3 (0.5%)
Not UIM (%)	356 (64%)
UIM (%)	196 (36%)
Semesters completed at start of clerkships	
4 or less (%)	408 (74%)
More than 4 (%)	144 (26%)
Total number of supervisors	4926
Total number of observations	24,187
By specialty	
Anesthesia	1295
Family and Community Medicine	3376
Internal Medicine	4185
Neurology	1846
Obstetrics/Gynecology	3211
Pediatrics	3258
Psychiatry	1978
Surgery	3866
Other/Unspecified	1172
By task type	
Communication	1908
History	3692
Note Taking	3707
Oral Presentation	6585
Physical Exam	2785
Procedure	2633
Other	2877
Entrustment-Supervision (ES) level rating distribution (%)	
1—Student required complete guidance or was unprepared	119 (0.5%)
2—Student was able to perform some tasks but required repeated directions	1674 (6.9%)
3—Student demonstrated some independence and only required intermittent prompting	8694 (35.9%)
4—Student functioned fairly independently and only needed assistance with nuances or complex situations	13,700 (56.6%)
Mean assessments per student [SD]	43.8 [33.9]
Mean assessments per supervisor [SD]	4.9 [8.8]
Mean words per feedback narrative [SD]	37 [21]
Mean sentences per feedback narrative [SD]	2.3 [1.3]
Mean entrustment-supervision (ES) level [SD]	3.1 [0.6]
Number of assessments documented by	
Student (%)	20,535 (84.9%)
Supervisor (%)	3652 (15.1%)

Of note, the majority of feedback dialogs were documented by trainees (20,535) while supervisors documented comparatively fewer (3652). To accommodate for this asymmetry in the data, as well the absence of simultaneous documentation by a supervisor and trainee of the same feedback dialog, we sought not to make comparisons of viewpoints from each dialog, but of dialogs representing similar task types in similar contexts. Thus, our multilevel statistical model included the task-related and contextual variables in Table 1 as covariates, and used student and supervisor identities to define clusters (described below).

### AI algorithms: language models to identify and measure entrustment-related themes and sentiment

*NLP-assisted theme extraction* (Fig. 1a)*.* We designed an NLP strategy to broadly characterize the set of themes in the overall narrative dataset (without considering which themes may or may not relate to entrustment), and then measured how strongly each theme was reflected in each feedback narrative. To characterize the themes, we utilized the Universal Sentence Encoder (USE) by Google, Inc. to represent the thematic content of each narrative numerically (Cer et al., 2018). The USE is a language model designed to compare the meaning of sentences and paragraphs by encoding them as a vector embedding—a 512-dimensional vector whose dimensions abstractly represent semantic meaning. We applied the USE to each feedback narrative in our dataset, generating a vector embedding representing each narrative. We then applied PCA to the set of standardized^3^ vector embeddings generated by the USE from all narratives in our dataset to identify the strongest principal components (dimensions) contributing to our dataset’s overall thematic variance (Joliffe & Morgan, 1992). We retained the first 17 principal components, which represented 33% of the overall thematic variance.

The qualitative themes represented by the principal components need to be identified based on the subsets of narratives associated with each component. To accomplish this, we empaneled a group of human coders with backgrounds in medical education (authors BG, CB, PO’S, and OtC) to perform thematic analysis on each of the 17 principal components. Mirroring the PCA coding procedure we developed and described in the appendix of our prior work (Gin et al., 2022), we identified the subsets of narratives that most strongly projected onto both directions of every principal component (i.e. the 99th and 1st percentiles), and found that they did indeed represented coherent themes. Thus, we were able to perform standard reflexive thematic analysis to code each subset of narratives separately (i.e. two subsets for each principal component). Each subset was coded independently by at least two coders, and we iterated until we reached consensus on the themes represented by the two directions of each principal component (Supplemental Table S2). Once the themes were verified, we conducted regression analysis to assess the correlation of these themes with entrustment rating (discussed below).

*Thematic reflexivity and NLP algorithmic considerations.* Our overall approach to coding could thus be viewed as a hybrid between NLP-assisted topic modeling combined with human-based coding of those topics (D. Zhang et al., 2016). The reflexivity and positionality considerations discussed above are thus reflected in our coding of each factor (Cambo & Gergle, 2022; Gin, 2023). “Algorithmic reflexivity” would be represented here by the choice of principal components representing the dataset. For example, employing a different LLM embedding than the USE (for example, using GPT-3/4 embeddings instead) may yield different principal components (Balkus & Yan, 2023). While other dimensional reduction techniques could also be employed, we chose to employ PCA here instead of other clustering techniques (such as gaussian clustering or HDBSCAN) after first testing those other algorithms (Malzer & Baum, 2020). The PCA analysis had an advantage over other techniques in producing coherent themes consistently with our dataset. Finally, we opted not to use newer LLMs here such as GPT-4, LLaMA 2, or Falcon (which were available at time of writing) because even the largest of these models was restricted by a token limit (2^15^ tokens, or about 25,000 words for GPT-4) that would prevent them from considering the dataset in its entirety, as we could do with the stepwise strategy outlined here. Furthermore, use of shared cloud computing resources (often required by larger LLMs) would have violated our institutional security policy on the use of sensitive data. Conversely, we did not use more traditional NLP methodologies such as TF-IDF or other bag-of-words based techniques, since these strategies are based on word frequency only, without consideration for the meaning of patterns or sequences of words (Agarwal & Nayak, 2020).

*Approach to gender-neutral sentiment analysis*. Sentiment analysis is the practice of assigning emotional valence to narrative data and is a field at the intersection of linguistics and machine learning with broad applicability to academic, commercial, and educational purposes (Nandwani & Verma, 2021). Sentiments may be as varied as multiple emotional axes or simply construed as positive versus negative. For our study, the goal of sentiment analysis was the latter—to estimate the probability that each feedback narrative had a positive emotional valence (compared to a negative one).

To perform our sentiment analysis (Fig. 1b), we started with BERT-PubMed—a specialized version of the LLM, BERT, which was trained on text from MEDLINE/PubMed (Devlin et al., 2019). We placed BERT-PubMed as the encoding layer in a LLM classifier whose output was a numerical probability (from 0 to 100%) that its input text reflected a positive versus negative sentiment of the writer. We then trained this sentiment classifier to predict sentiment using the Stanford Sentiment Treebank (SST-2), a collection of 11,855 sentences extracted from movie reviews annotated by human judges (Socher et al., 2013), or the Large Movie Review Dataset 1.0 (LMRD) a collection of 50,000 individually labeled (as positive or negative) movie reviews selected for their polarity (Maas et al., 2011). The annotated python source code we used for training (including LLM training details) is available in the online repository given in the above footnote.^2^ After training the LLM, we applied it to each narrative in our dataset, generating a probability for each narrative. We found that regardless of which training dataset we used (either the SST-2 or LMRD), there was a substantial negative bias in the sentiment probabilities when the pronouns were female (approximately − 5% probability of being positive) or gender-neutral (approximately − 10%), compared to when the pronouns were male.

*Mitigating algorithmic bias.* In order to mitigate this apparent gender bias in our sentiment classifier,^4^ we replaced all gendered pronouns with their gender-neutral equivalents in the LMRD training dataset, and re-trained the LLM (we opted to train using only this modified LMRD dataset for the final analysis, since it represented a larger collection of narratives). We also replaced all gendered pronouns from our narrative dataset before analyzing it (Bhardwaj et al., 2021). We then applied the trained LLM to each gender-neutral feedback narrative in our dataset (excluding other variables such as entrustment rating or demographics), generating a probability for each narrative of its positive sentiment (thus, a probability 100% represents positive sentiment and 0% represents negative sentiment, while 50% represents neutral sentiment).

*Computation, Data security, and Ethical Review*. We performed all AI modeling using TensorFlow 2.10 in Python 3.9 (Abadi et al., 2015), with all computation running entirely locally on a secured entry-level consumer computer with a discrete graphics processing unit which did not have any specialized capability. We performed all statistical analysis locally as well, using Stata 17.0 (Rabe-Hesketh & Skrondal, 2012). Thus, we maintained security of the data (and hence, anonymity of participants) without exposing it to any cloud/online or shared computing resources. To additionally de-identify the data prior to its use in the study, the identity of each participant was removed and replaced with a random token by a third party not associated with this study. Furthermore, we did not use any participant data to train the AI algorithms (Masters, 2023); all training data was derived only from public datasets. Our Institutional Review Board reviewed the ethical considerations of our study and approved the study protocol (study ID 20-32478).

### Statistical analysis

*Examining the relationship between feedback themes and entrustment* (Fig. 1a)*.* To determine how strongly each theme related to the assignment of entrustment ratings, we performed a multilevel multivariable ordinal logistic regression using the entrustment rating (trust_level), as the dependent variable, and the 17 (standardized) PCA-derived components of each feedback narrative (PCA_0-PCA_16) as the independent variables using Stata’s meologit function: 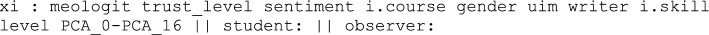


We included the following confounders/covariates in the model: clerkship rotation specialty (course), task type (skill), number of semesters completed by the student at the start of their rotations (level), student gender (gender), student UIM status (uim), sentiment of the narrative (as discussed above) (sentiment), and whether the feedback narrative was documented by the student or their supervisor (writer). The multilevel structure accounted for non-independence (multiple feedback dialogs) of observations related to each student by clustering observations by de-identified student (student:), and the identities of the supervisors (observer:) within each student cluster as a bi-level multilevel model.

The magnitude of the logistic regression coefficient between the entrustment rating and each theme’s associated PCA projection thus reflected how strongly that theme independently influenced the entrustment rating (positive coefficients related to the theme coded from the 99th percentile of narratives projecting onto the given principal component, while negative coefficients related to the theme coded from the 1st percentile of narratives) (Gin et al., 2022).

*Comparing viewpoints and assessing for bias* (Fig. 1a)*.* To compare student and supervisor viewpoints of the identified themes, we added interaction terms (to the above regression) between the feedback writer and each factor’s representative PCA projection (writer#c.(PCA_0-PCA_16)) (Rabe-Hesketh & Skrondal, 2012):
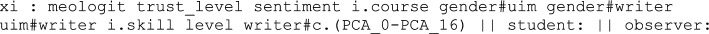


The correlation coefficients specific to the set of students and supervisors could thus be identified. Similarly, to assess for bias related to demographics, we added interaction terms between the feedback writer, the student’s gender identity (male vs female only, as there was not enough data to draw conclusions from observations of students identifying as neither male nor female), and UIM status (gender#uim gender#writer uim#writer). 

To assess for differences between sentiment and entrustment ratings based on writer role (supervisor vs. trainee), gender, and UIM status, we first conducted multilevel multivariable linear regressions for sentiment and entrustment rating (using the same covariates/confounders as the ordinal logistic regression described above) and then computed the estimated marginal means from the fully fit models (using the xtmixed and margins functions, respectively):
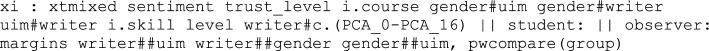



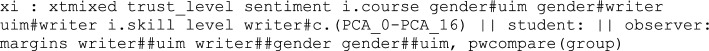


(This strategy for comparing average sentiment is shown in Fig. 1b, while the analogous procedure for average entrustment rating is not shown.) We assessed for statistically significant differences between groups using pairwise comparisons at the *p* < 0.05 level (using the pwcompare by group method).

## Results

We present our findings by the three elements of our research question, with our focus on understanding how trainee and supervisor perspectives differ when engaging in feedback about entrustment to perform a clinical task. First, we examine how trainees’ and supervisors’ documentation of the feedback dialog differed with respect to how feedback themes correlated with the entrustment rating, giving insight into factors trainees and supervisors viewed as important for entrustment. Secondly, we examine how the sentiment of the narratives differed between documentation written by supervisors and trainees, giving insight into how the language of feedback may reflect different attitudes of supervisors and trainees towards learning and improvement. Lastly, we examine how potential biases related to trainee gender and UIM may affect both the use of language and the assignment of entrustment levels, comparing documentation by supervisors and trainees to assess whether biases may differentially affect each perspective.

### Trainees vs supervisors: Which feedback themes correlate with entrustment ratings?

We identified and ordered feedback themes (principal components) with a statistically significant correlation to the entrustment rating by the strength of that correlation (as measured by the logistic regression coefficient β and odds ratio), doing so separately for supervisors and trainees (Table 2). We included only the themes with coefficients β > 0.10 (i.e. an odds ratio > 1.1) and *p*-values < 0.02. Each theme in the table thus represents features of a trainee’s clinical performance that are associated with higher levels of entrustment, with the importance of those themes to entrustment reflected in their ordering from top to bottom.

**Table 2:** Feedback themes demonstrating the strongest association with entrustment ratings, documented either by supervisors or trainees

Supervisors	Trainees
**Oral presentations were concise, thorough, and/or organized β = 0.31 (0.05), OR 1.37**	**Oral presentations were concise, thorough, and/or organized β = 0.47 (0.03), OR 1.60**
**Communications with patient were effective β = 0.25 (0.05), OR 1.29**	Nonspecific praise β = 0.29 (0.03), OR 1.34
Presentations included relevant details β = 0.14 (0.04), OR 1.16	**Communications with patient were effective β = 0.18 (0.03), OR 1.20**
Presentations included proper sections β = 0.14 (0.05), OR 1.15	Suggestions for improving clinical reasoning β = 0.14 (0.02), OR 1.16
Assessments were thorough β = 0.13 (0.04), OR 1.14	Asked appropriate questions β = 0.14 (0.02), OR 1.15
Suggestions for improving history of present illness (HPI) β = 0.12 (0.05), OR 1.12	Physical exams were comprehensive and relevant β = 0.13 (0.03), OR 1.14

Association strength is measured as the logistic regression coefficient β (with standard error given in the parenthesis, followed by the odds ratio, OR) for the entrustment rating as predicted by each theme’s standardized PCA components, over narratives in the entire dataset. The bold cells represent pairs of themes of high relevance to trust for both supervisors and trainees. We included here only the themes with coefficients > 0.10 and *p*-values (not shown) < 0.02

Although supervisors and trainees were equally empowered to document their shared feedback dialog, only two out of the top six themes matched when comparing documentation by supervisors and trainees. Effective patient communications and oral presentations were the two themes that correlated with entrustment ratings in both supervisors’ and trainees’ documentation. In supervisors’ documentation, feedback themes correlating with increased entrustment were heavily dominated by the oral presentation’s structure, organization, length, and inclusion of relevant detail. In trainees’ documentation, feedback associated with higher entrustment appeared to encompass a wider variety of themes, including general praise, asking appropriate questions, physical exam skills, and suggestions for improving clinical reasoning.

### Trainees vs supervisors: How does the sentiment of feedback documentation differ?

**Fig. 2:**
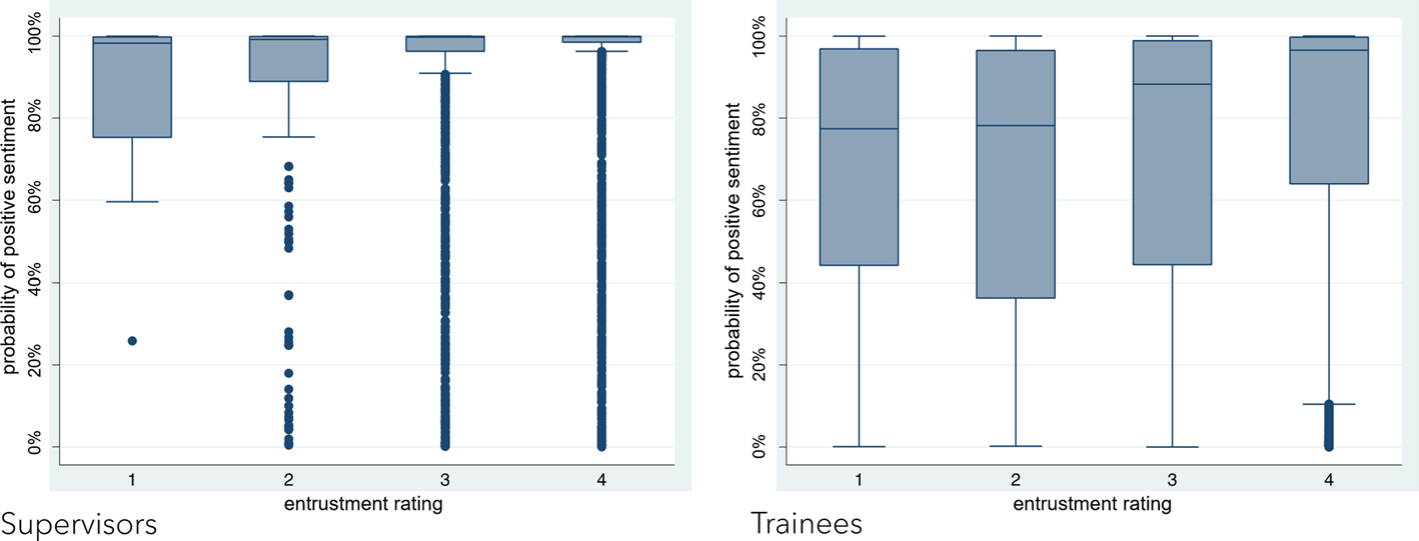
Box plots depicting the distribution of sentiments of feedback narratives. We trained an LLM to measure the sentiment of each feedback narrative as the probability of that sentiment being positive (scale of 0–100%, positive = 100%, negative = 0%). The narrative dataset was divided by writer (supervisor vs trainee) and then segregated further by entrustment rating. The distribution of sentiment in each of these subsets is depicted as a box plot showing the distribution mean (straight line), quartiles (box and whiskers), and outliers (dots)

Compared to supervisors, trainees tended to document feedback dialogs with language that utilized comparatively negative sentiment (− 5.3% ± 1.4% probability, based on a 95% confidence interval, CI) (distribution shown in Fig. 2). Direct examination of the feedback narratives revealed that sentiment was often used by supervisors to balance constructive feedback with praise (i.e. the proverbial “feedback sandwich”) (Parkes et al., 2013), while trainees appeared to focus on the constructive feedback more directly. The LLM tended to associate praise with positive sentiment, while constructive comments tended to be measured as negative. For example, the following narrative from a supervisor documented constructive feedback between phrases of praise:

Good job doing a comprehensive history and physical examination on a patient with exacerbation of congestive heart failure. I was impressed with the level of detail and the thoroughness of the presentation. As we discussed, I would start to think about what information needs to be a part of an oral presentation, versus what important information can simply be recorded in your written note for reference. This will help to make presentations more concise and easier to follow. Great start!

In comparison, a trainee’s documentation of the same skill type was more direct:

Overall, ***'s presentation of their patient during our coach-led preceptorship was comprehensive. Some points to work on: 1) non-pertinent information in HPI can be moved to ROS to streamline narrative and 2) organize medication list by diagnosis (will be helpful to listener and help them follow along).

We found the opposite trend when comparing average entrustment ratings between supervisors and trainees. Trainees appeared to recall a significantly higher level of trust (and lower level of supervision) when documenting feedback (0.08 ± 0.04 on a 1–4 entrustment scale) than supervisors did, which is consistent with results of other studies (Marty et al., 2021; Sterkenburg et al., 2010).

### How do gender and UIM status influence sentiment, entrustment ratings, and feedback themes?

We found that potential sources of bias (related to trainee gender and UIM status) appeared to affect sentiment (Table 3). We found five differences when examining sentiment written by supervisors versus trainees: On average, feedback written about male trainees tended to employ higher (more positive) sentiment than feedback about female trainees (1.3 ± 1.2%, *p* < 0.05). However, there was no significant difference in entrustment rating between female and male trainees (in fact, the average entrustment rating was identical). We examined whether supervisors or trainees were more prone to gender bias by looking for interactions between writer and gender variables. In the subset of narratives written by supervisors, there was no significant gender difference in either sentiment or entrustment level (i.e. its confidence interval of 1.4 ± 2.0% contained zero), while in the subset written by trainees, we found a significant gender difference in sentiment (1.3% ± 1.0%, *p* < 0.05), but not in entrustment ratings.

**Table 3:**
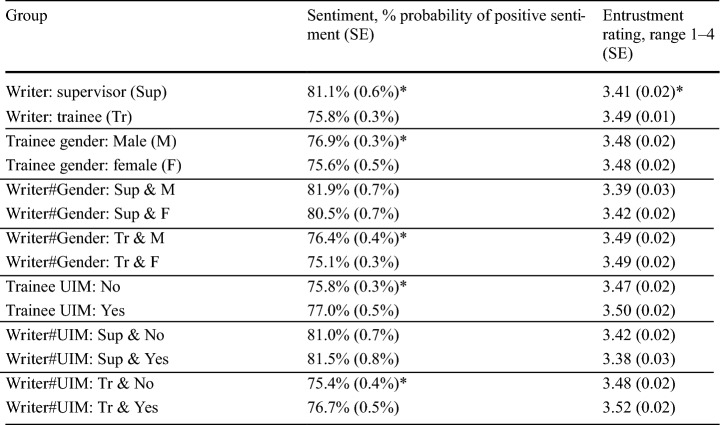
Pairwise comparison of average sentiments and entrustment ratings by group

Estimated marginal means from the linear multilevel models were applied to different groups (narrative writer, trainee gender, and trainee underrepresented in medicine (UIM) status), with standard errors given in the parentheses. The “#” represents statistical interactions between the indicated groups. Means that are significantly different *within* each paired comparison are marked with a “*” (significance at the *p* < 0.05 level). Significant differences *between* pairs are not shown

Finally, we compared the mean sentiment and entrustment ratings in the groups defined by trainee UIM status. We found that sentiment was more positive for UIM trainees (1.2 ± 1.1%, *p* < 0.05). However, this trend was only significant in feedback documented by trainees (1.3 ± 1.2%, *p* < 0.05), not in feedback documented by supervisors. We found no significant differences in mean entrustment ratings related to trainee UIM status.

Given the evidence of potential bias related to gender and trainee UIM status, we examined whether feedback themes may also be influenced by gender and UIM status (Table 4). Again ordering these themes by their correlation with entrustment ratings, we found that four out of the top six themes were the identical when comparing feedback written about male and female trainees. Of the four themes differing between groups, effective communications and presentations with relevant details were associated with higher trust in feedback written about male trainees, while properly sectioned presentations and comprehensive/relevant physical exams were associated with higher trust in feedback written about female students. We did not find significant differences in feedback themes partitioned by trainee UIM status.

**Table 4:** Feedback themes correlated with entrustment ratings by gender (in narratives written by both supervisors and trainees)

Male trainees	Female trainees
**Oral presentations were concise, thorough, and/or organized β = 0.41 (0.04), OR 1.51**	**Oral presentations were concise, thorough, and/or organized β = 0.47 (0.03), OR 1.60**
**Nonspecific praise β = 0.27 (0.04), OR 1.31**	**Nonspecific praise β = 0.24 (0.04), OR 1.27**
Communications with patient were effective β = 0.25 (0.04), OR 1.28	**Asked appropriate questions β = 0.15 (0.03), OR 1.15**
**Suggestions for improving clinical reasoning β = 0.16 (0.03), OR 1.18**	**Suggestions for improving clinical reasoning β = 0.13 (0.03), OR 1.14**
**Asked appropriate questions β = 0.14 (0.03), OR 1.15**	Physical exams were comprehensive and relevant β = 0.13 (0.03), OR 1.14
Presentations included relevant details β = 0.08 (0.03), OR 1.09	Presentations included proper sections β = 0.12 (0.03), OR 1.12

As in Table 2, themes in common between groups are bold

## Discussion

By employing an NLP strategy to perform theme extraction and sentiment analysis across a large dataset of documented feedback dialogs, we were able to identify trends in supervisor and trainee documentation that suggest differences in their cognitive and affective perspectives of entrustment. Further, our findings suggest that potential sources of bias derived from trainee gender identity and UIM status appear to affect the sentiment of documentation more so than the assignment of entrustment ratings. The ability to detect these small but statistically significant differences relied on consistent interpretation of narratives across a large dataset, for which we relied on LLM-based algorithms to augment the ability of human coders. Additionally, detection of small but significant biases in the sentiment writers expressed depended on mitigation of algorithmic bias, which would have masked the biases we wanted to investigate. While further investigation will be needed to assess the transferability of our findings, the methods we developed here could be utilized to quantitatively assess qualitative features of other large narrative datasets.

Using LLMs to augment qualitative coding, we were able to quantitatively investigate themes tied to entrustment ratings in feedback narratives documented by supervisors and trainees, giving insight into their cognitive decision-making around entrustment (Table 2). Both supervisor and trainee perspectives emphasized the importance of delivering effective oral presentations in determining entrustment. Both perspectives documented narratives that linked not only reinforcing feedback to entrustment, but also constructive feedback. Constructive comments about improving the HPI and clinical reasoning were also correlated positively with the entrustment rating. This finding suggests that open dialog may have been more important to building trust than the particular level of competence the trainee may have displayed (Castanelli et al., 2022; Telio et al., 2015). In terms of differences, while supervisors’ documentation tended to focus on presentations, trainees’ documentation expanded upon a broader scope of clinical skills and personal qualities. While this discrepancy may indicate patient presentations were central to supervisors’ interactions with trainees, it also may tie to trainees’ developing comfort within their roles in the clinical learning environment (Gruppen et al., 2019; O’Brien et al., 2007). Supervisors may have to make entrustment decisions primarily based on trainees’ presentations, but trainees need to consider a broader range of skills to be effective clinicians. An alternative explanation is that supervisors really do consider the patient presentation to be most reflective of trainee competence, since it involves the need to not only present data (for which effective physical exam and history taking skills would be a prerequisite) but also to synthesize it. This would provide empirical evidence to support the viewpoint that patient presentations represent a “signature pedagogy” of medicine (Gardner & Shulman, 2005; Irby, 1994). Further investigation is needed to clarify the importance of the patient presentation in how supervisors assess trainee capability when making entrustment decisions.

Our study revealed that the sentiment of documentation written by trainees was more negative on average compared to that written by supervisors, across all levels of entrustment (Fig. 2). Further investigation is needed to determine the source of this discrepancy. Some possibilities include that: (1) supervisors may intentionally omit negative language with the aim of maintaining their relationship with trainees, regardless of entrustment rating (Dudek et al., 2005; Ginsburg et al., 2016), and (2) supervisors may use positive sentiment to intentionally promote trainee acceptance of their feedback, i.e. the proverbial “feedback sandwich” (Sargeant et al., 2008). Additionally, a follow-up study could examine if a trainee’s emotional state may affect their prioritization of entrustment-related factors, potentially leading them to emphasize a broader range of skills than their supervisors (i.e. Table 2). While such a link between emotions and cognition has been suggested (Simon, 2020) and would be consistent with our findings, our data more concretely demonstrate that emotions are affected by biases related to trainee demographic characteristics.

The sentiment of trainees’ writing appears to have been biased by trainee gender and UIM status (Table 3). Female trainees appeared to use a more negative tone when documenting feedback dialogs than their male counterparts. UIM students appeared to use a more positive tone when documenting feedback. These differences in tone may reflect trainees’ perceptions of self-efficacy (Nomura et al., 2010; Sagasser et al., 2017)—which we did not explicitly assess (trainees were asked to document the feedback dialog they had with their supervisor, not to provide a self-assessment). Nevertheless, trainees’ self-efficacy may have shaped their acceptance of their supervisors’ feedback, and therefore their emotional response towards it. For comparison, several studies have found conflicting evidence of gender bias in feedback, as related to sentiment, assessment ratings, or clinical content. With respect to sentiment, Andrews et al. (2021) utilized NLP to analyze narratives in assessments of internal medicine residents, finding no significant difference between male and female subgroups. In our study, neither finding of bias (related to trainee gender or UIM status) was found in supervisors’ documentation, which may reflect an institutional culture promoting faculty consideration of diversity, equity, and inclusion (DEI) at the site we studied (Lucey et al., 2020; Teherani et al., 2020). For comparison, Sarraf et al. (2021) found significant gender bias in letters of recommendation written at their institution for general surgery residency candidates; but their sample included letters from decades before significant DEI efforts are likely to have been made.

Several studies have also examined whether the thematic content of feedback can be biased by trainee gender. Mamtani et al. (2022) compared themes in feedback given to male and female residents in emergency medicine, finding multiple differences in the frequency with which these themes were found. Female trainees were more likely to receive feedback related to their procedural confidence rather than competence. Their study examined the frequency of themes found in feedback, but did not consider how those themes were tied to a quantitative performance metric such as the entrustment rating. Here, we have considered not only the frequency of themes (factors), but also the degree to which they are correlated with the entrustment rating. This difference in methodology is specific to our research question of identifying factors related to entrustment, rather than the general scope of feedback. With this consideration in mind, our results revealed that documentation of feedback about male and female trainees was more similar than it was different, which mirrors the results found by Andrews et al. (2021) of feedback topics describing internal medicine residents. We found that feedback about male and female trainees emphasized both competency performance (i.e. qualities of the presentation, physical exam, and communications) as well as personal characteristics (i.e. asking appropriate questions). The main gender discrepancy we found was that feedback about male trainees included prioritization of communications skills, whereas for female trainees the physical exam was emphasized. Further investigation is needed to understand the significance of this difference. For comparison, Mamtani et al. (2022) similarly found that their male subgroup received feedback about communication skills with higher frequency.

Our finding that bias can affect entrustment-related feedback may relate to inherent vulnerabilities when making decisions based on swift trust. Swift (or initial) trust refers to trust that is created via first impressions, and thus heavily dependent on emotionally-driven and subconscious judgements (ten Cate et al., 2016). Ad-hoc entrustment decisions (which the encounters in our dataset represented) may depend on swift trust more than summative entrustment decisions, since they may be derived from infrequent encounters between supervisors and trainees, and/or encounters at early stages of relationship formation (Gomez-Garibello & Young, 2018). Swift trust contrasts with presumptive trust (based on credentials only, without prior interaction with an individual) and grounded trust (based on evidence collected over multiple interactions), and thus may be more susceptible to biases harbored unconsciously by the trustor (Hendren & Kumagai, 2019; Teherani et al., 2018). The evidence of bias we found in trainees’ documentation may thus not reflect trainee biases towards themselves, but rather their response to biases they perceive as being directed towards them—perhaps perpetuated by an unequal power differential that allows biased entrustment decisions based on swift trust to go unchallenged. Such biases may represent the undercurrents of unmitigated disparities in the clinical learning environment and warrant further scrutiny.

This study has limitations. As previously discussed, there was asymmetry in the number of feedback dialogs documented by trainees compared to supervisors. Rather than comparing trainee and supervisor documentation of the same feedback dialog, we made comparisons of viewpoints of similar types of clinical tasks in similar contexts by designing a multilevel model accounting for these variables. This strategy does not account for the possibility that there was selection bias as to whether the trainee or supervisor documented the feedback (i.e. supervisors may be more likely to document feedback if it is positive). However, the entrustment ratings’ apparent invariance to the potential sources of bias we investigated suggests that the effect of such a selection bias on entrustment ratings could also be small. Further, the generalizability/transferability of our data remains to be investigated, since we focused on a single institution with medical students as trainees. A study during residency may emphasize different factors as important for entrustment since the opportunities for trainee independence would be greater and the risk of harm to patients from inappropriate entrustment would potentially be higher. The NLP techniques we developed here could be readily applied to feedback obtained in such a setting. Finally, we note a methodological limitation that involves the scope of the knowledge transferred from the trained LLMs we used. All LLMs transfer inferences from training sets to the analysis of study data; as such, they are susceptible to biases and a lack of generalizability. We provided strategies here for mitigating such biases (i.e. the gender-neutral LLM design), and for improving generalizability: utilizing a panel of human coders to assess themes derived from the LLM, and combining a movie-review based sentiment training set with an LLM trained on PubMed.

We conclude that while bias persists in workplace-based assessment, it appears to influence the emotive language trainees use to document entrustment more than the degree of entrustment they experience. Trainees also considered a broader range of factors when rationalizing the level of entrustment they required, compared to supervisors who focused on trainees’ patient presentations. While the sentiment of trainees’ writing was biased by gender and UIM status, bias did not appear to influence the linked entrustment ratings, even when trainees documented those ratings. It is somewhat reassuring to find that entrustment ratings appeared to be less susceptible to bias, given their expanding uses in formative and summative assessment. While entrustment frameworks, AfL, and DEI-related interventions may be improving parity in assessment, the persistence of biases reflected in our data indicates there is still much opportunity to improve the inclusiveness of the clinical learning environment.

## Supplementary Information

Below is the link to the electronic supplementary material.

Supplementary file1 (DOCX 13 kb)

## Footnotes

1 In AI/ML terminology, “transfer learning” refers to employing algorithms trained on datasets external to the dataset being analyzed.

2 https://github.com/briancgin/entrustment-feedback.

3 Each dimension scaled and centered to have a standard deviation of 1 and mean of 0.

4 It is likely that the bias in the sentiment prediction derived from gender bias in the training datasets, but it is also possible that the underlying PubMed-trained BERT LLM also contained gender bias.
