# Supplementary material for: Assessing supervisor versus trainee viewpoints of entrustment through cognitive and affective lenses: an artificial intelligence investigation of bias in feedback
Source: Adv Health Sci Educ Theory Pract. 2024 Feb 23;29(5):1571–92. doi: 10.1007/s10459-024-10311-9 (PMC11549112; doi:10.1007/s10459-024-10311-9)
Supplement: Supplementary file 1 — Supplementary file1 (DOCX 13 kb) [file 10459_2024_10311_MOESM1_ESM.docx]

Supplemental Table S1: **The entrustment rating scale used**, based on the Modifed O-SCORE.

*Level 1*: “I did it.” Student required complete guidance or was unprepared; I had to do most of the work myself.

*Level 2*: “I talked them through it.” Student was able to perform some tasks but required repeated directions.

*Level 3*: “I directed them from time to time.” Student demonstrated some independence and only required intermittent prompting.

*Level 4*: “I was available just in case.” Student functioned fairly independently and only needed assistance with nuances or complex situations.

Supplemental Table S2: **Feedback themes identified from principal component analysis (PCA)** of the LLM embedding vectors. The first 17 principal components (PC) were retained. The themes were identified from the PCA analysis via a qualitative coding approach that included human expert opinion.

| **PC #** | **(+) Coefficient theme** | **(-) Coefficient theme** |
| --- | --- | --- |
| **0** | nonspecific praise | communications/rapport with patients |
| **1** | oral presentations were concise, thorough, and/or organized | constructive feedback on procedures, “make sure…” |
| **2** | communications with patient were effective | assessment/plan/notes constructive feedback |
| **3** | physical exam mentioned - some constructive, no praise | suggestions re: conversation/ communication/language w/ patients |
| **4** | reinforcing + constructive presentation feedback | assessment were thorough |
| **5** | physical exams were comprehensive and relevant | continue to practice |
| **6** | specific advice on exams, constructive | suggestions for improving HPI |
| **7** | asked appropriate questions | constructive feedback about A/P |
| **8** | closed vs open ended questions | notes include the important components of a patient’s history |
| **9** | receptivity to feedback in general (i.e. feedback orientation) | history organization |
| **10** | suggestions for improving clinical reasoning | procedures, needle, cap, port, laparoscopy |
| **11** | presentations include proper sections | presentation organization/problem |
| **12** | excellent/thorough history | A/P problems and organization |
| **13** | praise of history and presentation | thoroughness/detail of notes and differentials |
| **14** | praise of differential | A/P organization inclusion/exclusion |
| **15** | thoroughness of history and A/P | presentations included relevant details |
| **16** | pertinent positives and negatives | notes clear/concise/detailed |
